# Editorial: Gut Health: The New Paradigm in Food Animal Production

**DOI:** 10.3389/fvets.2016.00071

**Published:** 2016-08-31

**Authors:** Michael H. Kogut, Ryan J. Arsenault

**Affiliations:** ^1^USDA Agricultural Research Service, College Station, TX, USA; ^2^University of Delaware, Newark, DE, USA

**Keywords:** gut health, production animals, Chickens, Swine, Cattle, microbiome, mucosal immunity

**The Editorial on the Research Topic**

**Gut Health: The New Paradigm in Animal Production**

Optimal gut health is of vital importance to the performance of production animals. Gut health is synonymous in animal production industries with animal health. Although there does appear to be a direct relationship between animal performance and a “healthy” gastrointestinal tract (GIT), there is no clear definition for “gut health” that encompasses a number of physiological and functional features, including nutrient digestion and absorption, host metabolism and energy generation, a stable microbiome, mucus layer development, barrier function, and mucosal immune responses (1–8). The GIT is responsible for regulating physiological homeostasis that provides the host the ability to withstand infectious and non-infectious stressors (9–19). Understanding the interactions between these diverse physiological features emphasizes the extent of areas encompassed by gut health and the ability to regulate animal production. For our part, we will define gut health as the absence/prevention/avoidance of disease so that the animal is able to perform its physiological functions in order to withstand exogenous and endogenous stressors. Furthermore, worldwide public concerns about the production animal industries’ dependency on the use of growth-promoting antibiotics (AGPs) have resulted in the ban of AGPs by the European Union and a reassessment of their use in the United States. Thus, current research is focused on alternatives to antibiotics for sustainable food animal production (20).

A recent Research Topic in Frontiers in Veterinary Infectious Diseases was on gut health and wondering whether we should consider gut health as the new standard when considering animal production. The objective of this Editorial is not to review the literature on gut health in production animals, but, rather, it is our attempt to summarize findings of the 15 papers that were published within this Research Topic. Obviously, the Topic was not comprehensive in the production animal commodity reported, but it was a very good overview of the current status of the ongoing work in gut health and physiology within the veterinary community.

## Gut Microbiome

The complex gut microbiome is not a silent organ or a collection of passenger microorganisms; but rather, the intestinal microbial community represents active participants in vertebrate immunity and physiology. The gut microbiota confers health benefits to the host, including aiding in the digestion and absorption of nutrients, contributing to the construction of the intestinal epithelial barrier, the development and function of the host immune system, and competing with pathogenic microbes to prevent their harmful propagation (18, 21). Unlike the host genome, which is rarely manipulated by xenobiotic intervention, the microbiome is readily changeable by diet, ingestion of antibiotics, infection by pathogens, and other life events [Danzeisen et al.; Ballou et al.; Mon et al.; Malmuthauge et al.; (8)].

Antibiotics have a great effect on the host normal microbiota upsetting the balance and inducing a dysbiotic state (8). The use of sub-therapeutic doses of antibiotics in animal diets have been a common practice for promoting growth due to their ability to increase feed efficiency or preventing diseases. Danzeisen et al. used a sub-therapeutic concentration of penicillin to define beneficial members of the microbiome in turkeys that resulted in increased feed efficiency and enhanced growth. By identifying the specific bacterial populations responsible for improved performance, the authors hypothesize that these bacteria can then be used as probiotics.

The microbiome has a direct effect on the development and function of the mucosal immune system. Malmuthauge et al. found significant associations between the microbiome and the expression of genes regulating the mucosal barrier and innate immunity in neonatal cattle. Regional differences in the microbiome were associated with regional differences in innate immune gene transcription. Similar findings were described between the microbiome of broiler chickens and the expression of avian cytokine RNA transcripts (Oakley and Kogut). A negative correlation between pro-inflammatory cytokine genes and the phylum Firmicutes was found; whereas a positive correlation was identified with the pro-inflammatory cytokines and the phylum Proteobacteria.

Wigley and Ballou et al. asked the questions: what constitutes a normal or healthy microbiome and what effects do treatments that are being used to improve gut health (vaccines and probiotics) have on the development of the gut microbiome? Wigley pointed out that certain bacteria, such as *Escherichia coli, Clostridium perfringens*, and *Campylobacter*, are often considered commensals and part of the cecal microbiome. The removal of AGPs, manipulation of the cecal microbiome, changing husbandry practices, and other internal and external factors lead to changes in the host responses that result in “new” infections (22–25). Using a live attenuated *Salmonella* vaccine or a lactic acid bacteria probiotic, Ballou et al. characterized the effects of gut health treatments have on the microbiome. Alterations in microbial diversity in the microbiome of young chicks given the vaccine and, to a less extent with the probiotic, were found, which were independent of bacterial colonization by the treatments. The microbiome alterations were maintained through 28 days of age, suggesting that early exposure to certain bacteria may permanently influence the microbial diversity in the microbiomes. Similar results were described by Mon et al. where a *Salmonella* infection in day-old chicks induced a profound decrease in microbial diversity in the cecum. Specifically, there was an increase in *Enterobacteriaceae* and a decrease in butyrate-producing bacteria in the *Lachnospiraceae* family implying that exposure to a *Salmonella* infection early after hatch can impact the composition of the developing microbiome that affects colonization resistance to microbial pathogens.

Yeast-derived dietary supplements are increasingly being used as pre- and probiotics to improve gut health (26). Roto et al. detailed the effects of yeast-derived compounds in livestock diets and their effect of the microbiome. The use of yeast-derived compounds as supplements in livestock diets improved performance, increased beneficial bacteria in the microbiome, and increased immune responsiveness. Additionally, the yeast-derived products are cost-effective, do not induce antimicrobial resistance in pathogens, and, because of their multiple mechanisms of action, can be used in the variety of environments found in livestock industries.

## Mucosal Immune Response

The intestinal tract is an active immunological organ with more resident immune cells than anywhere else in the body. They are organized in lymphoid structures called Peyer’s patches and isolated lymphoid follicles, such as the cecal tonsils. Macrophages, dendritic cells, various subsets of T cells, B cells, and secretory IgA all contribute to the generation of a proper immune response to invading pathogens, while keeping the resident microbial community in check without generating an overt inflammatory response.

In addition to the immune cells, the intestinal epithelial cells contribute to mucosal immunity (21). A single layer of epithelial cells separates the densely colonized and environmentally exposed intestinal lumen from the sterile subepithelial tissue, maintains homeostasis in the presence of the enteric microbiota, and contributes to rapid and efficient antimicrobial host defense in the event of infection with pathogens. Both epithelial antimicrobial host defense and homeostasis rely on signaling pathways induced by innate immune receptors demonstrating the active role of epithelial cells in the host–microbial interplay. Lastly, a layer of mucus overlying the intestinal epithelium forms a physical barrier between the mucosa and the resident microbiota, minimizing both microbial translocation and excessive immune activation by the resident microbes.

Intestinal integrity is fundamentally important for the growth and performance of food animals. One of the main advantages of AGPs in animal feed was the reduction in the low-grade, food-induced chronic inflammation that would otherwise be detrimental to animal growth (27). Removal of AGPs from animal feeds results in an increase in enteric disorders, infections, and diseases (24, 25, 28, 29). One of the issues with determining dysfunction of the gut barrier is the lack of specific biomarkers. Two papers in the Research Topic described new methods that: (a) identify serum and tissue biomarkers of gut barrier function (Chen et al.) and (b) identify a non-invasive means to measure gut inflammation as a marker of gut leakage (Kuttappan et al.). Additionally, Ayoola et al. found that the addition of supplemental enzymes (β-mannanase, a blend of xylanase, amylase, protease) to the diet of turkeys reduced food-induced inflammation.

One of the main immune functions of the epithelial cell surface is the production of antimicrobial peptides or host defense peptides [HDPs; Ref. (30)]. HDPs are a diverse group of small molecules that possess antimicrobial, immunomodulatory, and barrier function enhancing activities. Robinson et al. described several classes of small-molecule compounds that induce specific induction of endogenous HDP. Furthermore, supplementation of these HDP-inducing compounds enhanced bacterial clearance, improved enteric barrier integrity, and improved animal production efficiency with minimal intestinal inflammation.

The host/pathogen interactome leads to a number of immune and biochemical changes at the infection site as the pathogen tries to derive nutrients from the host, while the host uses immunometabolic countermeasures against the pathogen. Arsenault and Kogut developed a novel tool that characterizes the immunometabolic phenotype of infected cells/tissues. The kinome peptide array identifies alterations in phosphorylation events in both immunity and metabolism simultaneously. The kinome array was used to identify the immune changes occurring in the cecum of chickens during the establishment of a persistent, asymptomatic *Salmonella* infection (Kogut and Arsenault). A number of immune signaling pathways were activated at the site of infection that indicates the development of a tolerogenic response allowing the bacteria to establish a persistent infection.

## Direct Fed Microbials

The increased use of grains as alternative energy sources in poultry diets has led to an issue with higher levels of less digestible carbohydrates that result in an increase in digesta viscosity and food-induced inflammation. One alternative to optimize digestibility of these complex carbohydrates is the inclusion of dietary enzyme supplements. Latorre et al. took this concept a step further and described the selection of a *Bacillus* spp. direct fed microbial (DFM) candidate based on their capacity to produce enzymes that breakdown these complex carbohydrates. *Bacillus* spp. that produced cellulose and xylanase were used as DFM and were found to reduce digesta viscosity and reduce *C. perfringens* growth in a number of different diets containing different complex carbohydrates.

A group of natural products known as phytobiotics have been the focus of several studies in recent years as antibiotic alternatives (31). Phytobiotics are plant-derived products used in feed that possess antimicrobial activity, provide antioxidative effects, enhance palatability, improve gut functions, and promote growth. Murugesan et al. compared the effects of a commercial phytogenic feed additive on growth, intestinal morphology, and microbial composition in chickens to the effects of an AGP. Improved growth, increased intestinal villus height, and decreased total cecal numbers of *Clostridium* and anaerobic bacteria were comparable between the two treatments. However, birds fed the phytobiotic additives had a significant reduction in coliforms and an increase in *Lactobacillus* spp. implying an environment that was more suitable for the establishment of growth-promoting bacteria in the microbiome.

Although the GIT is frequently described simply as “the gut,” it is actually made up of (1) an epithelium; (2) a diverse and robust immune arm, which contains most of the immune cells in the body; and (3) the commensal bacteria, which contain more cells than are present in the entire host organism. Understanding of the crosstalk between ALL of these interrelated components of the gut is what cumulatively makes the gut the basis for the health of animals and the motor that drives their performance.

## Author Contributions

All authors listed have made substantial, direct, and intellectual contribution to the work and approved it for publication.

## Conflict of Interest Statement

The authors declare that the research was conducted in the absence of any commercial or financial relationships that could be construed as a potential conflict of interest.
